# Environmental predictors of bovine *Eimeria* infection in western Kenya

**DOI:** 10.1007/s11250-016-1209-0

**Published:** 2017-01-04

**Authors:** D. N. Makau, G. K. Gitau, G. K. Muchemi, L. F. Thomas, E. A. J. Cook, N. A. Wardrop, E. M. Fèvre, W. A. de Glanville

**Affiliations:** 1 Department of Public Health Pharmacology and Toxicology, Faculty of Veterinary Medicine, University of Nairobi, Nairobi, PO BOX 29053-00625, Kenya; 2 Department of Clinical Studies, Faculty of Veterinary Medicine, University of Nairobi, Nairobi, PO BOX 29053-00625, Kenya; 3 International Livestock Research Institute, Old Naivasha Road, PO Box 30709-00100, Nairobi, Kenya; 4 Centre for Immunity, Infection and Evolution, Institute for Immunology and Infection Research, School of Biological Sciences, Kings Buildings, University of Edinburgh, West Mains Road, Edinburgh, EH9 3JT UK; 5 Geography and Environment, University of Southampton, Highfield Campus, University Road, Southampton, SO17 1BJ UK; 6 Institute of Infection and Global Health, University of Liverpool, Leahurst Campus, Neston, CH64 7TE UK

**Keywords:** Eimeria, Bovine, Tropical, Environmental, Extensive production systems

## Abstract

Eimeriosis is caused by a protozoan infection affecting most domestic animal species. Outbreaks in cattle are associated with various environmental factors in temperate climates but limited work has been done in tropical settings. The objective of this work was to determine the prevalence and environmental factors associated with bovine *Eimeria* spp. infection in a mixed farming area of western Kenya. A total of 983 cattle were sampled from 226 cattle-keeping households. Faecal samples were collected directly from the rectum via digital extraction and analysed for the presence of *Eimeria* spp. infection using the MacMaster technique. Individual and household level predictors of infection were explored using mixed effects logistic regression. The prevalence of individual animal *Eimeria* infection was 32.8% (95% CI 29.9–35.9). A positive linear relationship was found between risk of *Eimeria* infection and increasing temperature (OR = 1.4, 95% CI 1.06–1.86) and distance to areas at risk of flooding (OR = 1.49, 95% CI 1.17–1.91). There was weak evidence of non-linear relationship between *Eimeria* infection and the proportion of the area around a household that was classified as swamp (OR = 1.12, 95% CI 0.87–1.44; OR (quadratic term) = 0.85, 95% CI 0.73–1.00), and the sand content of the soil (OR = 1.18, 95% CI 0.91–1.53; OR (quadratic term) = 1.1, 95% CI 0.99–1.23). The risk of animal *Eimeria* spp. infection is influenced by a number of climatic and soil-associated conditions.

## Introduction

Eimeriosis affects a wide range of animal species and is caused by infection with protozoa in the *Eimeria* genus, previously known as *Coccidia*. Eimeriosis in cattle is characterised by diarrhoea, fever, anorexia, weight loss, emaciation and sometimes death, particularly in young animals (Coetzer and Justin 2004). Transmission occurs following ingestion of sporoblasts in the environment. These undergo the first and second stages of schizogony in the small and large intestines, respectively, before gametogony in the colon. Oocysts are then passed in faeces to the environment where sporogony takes place and infection of another host can occur after ingestion of these oocysts with sporoblasts (Urquhart et al. 1996). Outbreaks of the disease have previously been found to be associated with environmental stressors, including low temperatures during cold seasons (Rodríguez et al. 1996; Maas 2007). A number of studies have also looked at management-related factors influencing *Eimeria spp.* infection in cattle. These include housing system, feeding system, watering system, floor type and herd size (Khan et al. 2013). Environmental conditions in cattle sheds, which influence the survival and sporulation of infective oocysts, have also been shown to influence risk of infection (Lassen et al. 2009).

While management factors and very cold weather conditions have been shown to be associated with increased clinical disease in herds in temperate settings, there has been relatively little research on the effect of environmental factors on the epidemiology of eimeriosis in the tropics. In a study done in Zimbabwe to assess the effects of season on gastrointestinal parasitism in cattle, Pfukenyi et al. (2007) reported that high rainfall was significantly associated with high OPG counts of *Eimeria* parasites. In another study in South Africa, Matjila and Penzhorn (2002) reported that the prevalence of *Eimeria* infection was highest in dairy farms that were nearest to the Pienaars River.

Soil composition has also been observed to influence oocyst recovery rate and identification (Lélu et al. 2011), with similar effects observed for a number of other protozoal parasites such as *Cryptosporidium* and *Toxoplasma* (Lélu et al. 2011).

The aim of this study was to determine the prevalence of bovine *Eimeria* spp. infection in cattle in a small-holder farming area of western Kenya and to assess individual and herd level factors associated with infection, with a particular focus on identifying environmental predictors.

## Methods

### Study area

**Fig. 1:**
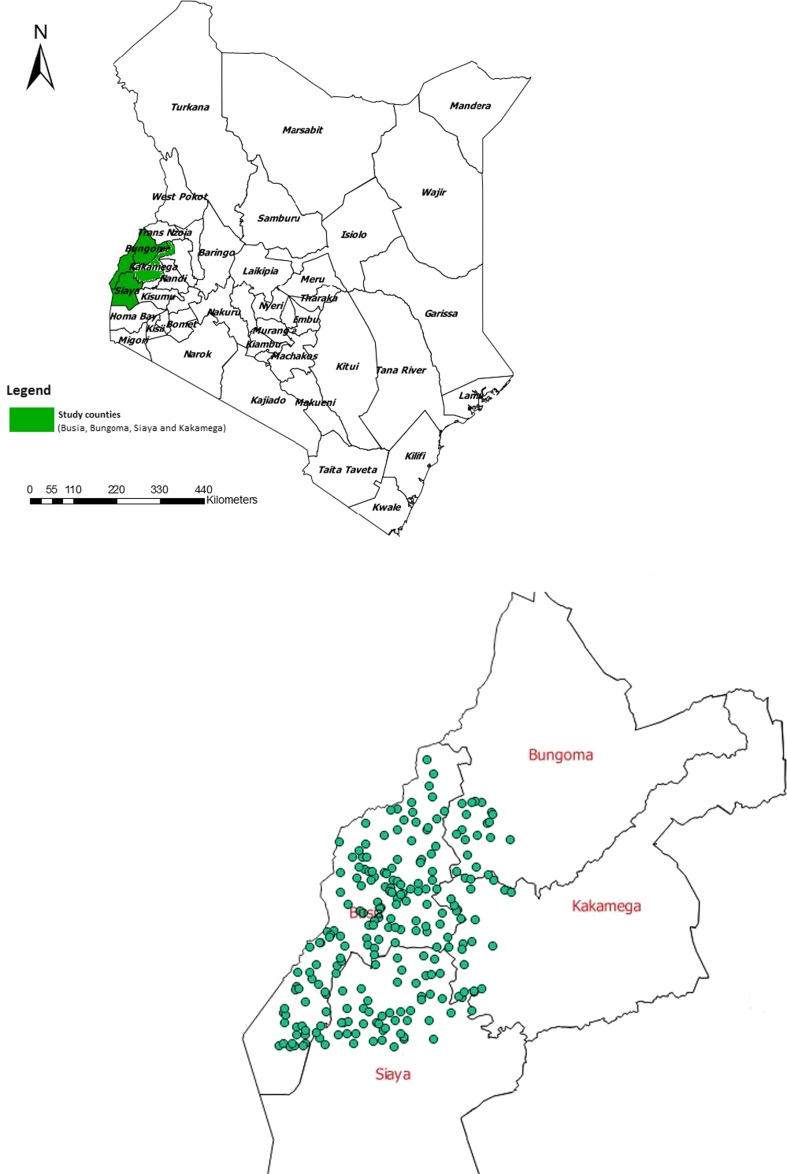
Study area in western Kenya, including location of sampled households

All data were collected as part of the People, Animals and their Zoonoses (PAZ) project between August 2010 and July 2012 (Doble and Fevre 2010). The study area was in Busia, Bungoma, Siaya and Kakamega counties in western Kenya (Fig. 1). This is an area with a predominantly small-scale, mixed-farming system, in which livestock production is integrated with agricultural production. The median cattle herd size is five animals of predominantly short-horn zebu breed, with animals typically managed using a mixture of tethered and free grazing systems on communal grazing lands (Bronsvoort et al. 2013).

### Ethical approval

Ethical approval for the animal elements of the study was provided by the Animal Welfare and Ethical Review Body of the Roslin Institute, University of Edinburgh, UK (AWA004).

### Sample collection

The PAZ study was focused on zoonotic pathogens in people and animals and was designed to detect the lowest expected prevalence of 5% in the bovine population with 2% standard error at the 95% confidence interval and a design effect of 3. A multistage sampling approach was adopted, with households randomly selected within sublocations, the smallest administrative unit in Kenya. All sublocations within 45 km of the study field station, located in Busia town, were included. Between 1 and 8 households were selected in each sublocation, with the number selected proportional to the cattle population present in the sublocation. Household selection involved the generation of an appropriate number of random points in each sublocation using a geographic information system (ArcMap 9.1, ESRI, Redlands CA). The closest consenting household that was within 300 m of the actual location of the random point was then selected for inclusion. A back-up point was used when there was no household within 300 m. Both livestock and non-livestock keeping households were included in the PAZ study. In each randomly selected household, a comprehensive household-level questionnaire was administered and data on herd profile, husbandry and management were obtained. All cattle within selected households were sampled, excluding those in the last trimester of pregnancy. Faecal samples were collected directly from the rectum via digital extraction. All faecal samples were analysed using the MacMaster technique (Levecke et al. 2012) to determine the presence or absence of *Eimeria* spp. oocysts. An individual was considered positive if oocysts were observed under microscopic examination.

### Predictors of *Eimeria* infection

Environmental predictors of *Eimeria* spp. infection considered a priori to be important in this setting were the proportion of a 1 km area around a household that was classified as crops and grassland; the proportion of the same area around a household classified as swamp land; household distance from water bodies (Wardrop 2015); distance from areas at risk of flooding; average maximum green vegetation fraction (MGVF) (Broxton et al. 2014); average temperature; average precipitation (Hijmans et al. 2005); and soil sand content (Hengl et al. 2013). Environmental data were derived for the study area from a range of sources, as described by Wardrop et al. (2015). The average and range of values for these environmental variables are presented in Table 1. Sampling for PAZ was carried out continually for 2 years, and to explore rainfall mediated seasonal effects on *Eimeria* infection, we also derived values for the average rainfall measured in the 30 days prior to the sampling visit to a household from the nearest weather station with consistent data (Kisumu, latitude = −0.09, longitude = 34.73, www.wunderground.com). Household geographic position and altitude were derived using a handheld GPS (Garmin eTrex). Household grazing system (zero or tethered grazing vs. free grazing) was extracted from the household questionnaire. Age based on dentition (dichotomised to <18 months and ≥18 months), sex and breed (local zebu vs. pure breed exotic dairy cattle and their crosses) were included as predictors at the individual level. All continuous variables were examined for outliers, and the different scales of each were standardized by subtracting the mean and dividing by the standard deviation. Correlation between continuous variables was assessed using Spearman’s rank test, with one of a highly correlated pair (≥0.7) excluded based on an assessment of the biological importance of each of a correlated pair. Correlations were explored using base commands in *R* version 2.1.1. (http://cran.r-project.org/).

**Table 1:** Summary of environmental determinants analysed in four counties in western Kenya

Factor	Average	Range
Percentage crop or grass land in 1 km area surrounding household	60.68%	35.83–78.10%
Percentage swamp land in 1 km area surrounding household	7.48%	0–36.41%
Soil sand content	42.39%	22–51%
pH of surface water	5.6	5.2–6.3
Temperature	22.1 °C	21.2–22.8 °C
Distance to water body	2826 m	88–9305 m
Distance to areas at risk of flooding	985 m	0–5636 m
Average MODIS-based maximum green vegetation fraction (MGVF).	86%	77–93%
Altitude	1225 m	1127–1405 m
Precipitation	1532 mm	997–1837 mm
Average rainfall in the previous 30 days	1.3 mm	0–6.0 mm

### Covariate relationships with *Eimeria* infection

Potential non-linear relationships between the log odds of *Eimeria* infection and each continuous covariate were explored using the method described by Hosmer et al. (2013). For this, a categorical variable with four levels was created using quartile cut-points. A logistic regression model was then fit replacing the continuous predictor with its four-level categorical derivative, and the resulting coefficients plotted versus the mid-points of the upper three quartiles (with the lowest used as reference, and given a value of 0). Where the relationship was obviously linear, the continuous form of the variable was used for inference. Where a logical parametric shape (e.g. quadratic) could be observed, made biological sense, and resulted in model improvement based on Akaike’s information criterion (AIC), the variable was transformed. Where the relationship appeared more complicated, a restricted cubic spline (RCS) with three knots was used. If the resulting fitted shape made biological sense and resulted in lower AIC when compared to the model containing the variable in its linear form, the RCS (defined using default cut-points (Harrell 2001, 2015) was used for inference. Restricted cubic splines were fit using the *rms* package in *R* (Harrell 2015).

Continuous environmental variables transformed according to this approach were included together with categorical predictors of infection (age, sex, breed, herd management) in a full logistic regression model that included household as a random effect. The model was fit using the *lme4* package in *R* (Bates et al. 2015). Model simplification was performed using the purposeful selection procedure described by Hosmer et al. (2013). Variables were removed from the full model in a step-wise manner if they were not significant and not a confounder. The significance threshold was set at the 0.1 alpha level, and confounding was assessed on the basis of a 20% or greater change in the value of the coefficient of any of the remaining variables following removal (Bursac et al. 2008). The final model was assessed for evidence of multicollinearity using variance inflation factors.

### Spatial analysis

A spatial scan statistic using a weighted normal model was implemented in SatScan version 9 (www.satscan.org) to detect spatial clustering in *Eimeria* infection risk in the study area. The statistic was estimated using household level residuals extracted from a null logistic regression model containing only the intercept and household level random effect. The model was weighted using the number of cattle sampled in each household (Alton et al. 2013). A normal model was used with 999 iterations (allowing estimation of *p* values down to 0.001), and a cluster size up to a maximum of 50% of observations. Household level residuals from the final logistic regression model derived from the purposeful selection procedure described above were also examined for evidence of residual spatial autocorrelation using spline correlograms using the *ncf* package (Bjornstad 2016).

## Results

### Summary statistics

A total 416 households were recruited into the PAZ study. Of these, 226 had cattle and 983 individual animals were included. From these, 93% of animals had faecal samples collected. The study population was 66% female while Zebu breeds and short horn zebu crosses made up 77.4%. A total of 295 (30.0%) of the animals sampled were less than 18 months.

The prevalence of individual animal *Eimeria* infection was 32.8% (95% CI 29.9–35.9%). The median oocysts per gram (OPG) was 200 while the average OPG was 1447.

### Model selection

High levels of correlation were observed between altitude and temperature (*r* = 0.97). Since the effect of altitude on *Eimeria* risk could be expected to be mediated through temperature-associated effects on oocyst sporulation, temperature was retained in the model.

The inclusion of a quadratic term improved the fit (based on AIC, data not shown) of the proportion of the area around a household that was classified as swamp and the sandy content of soil. All other environmental predictors were included in their linear form.

**Fig. 2:**
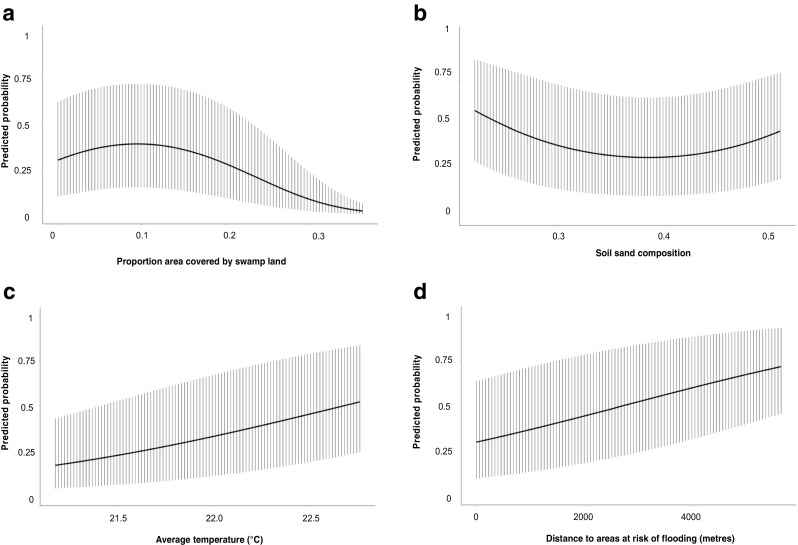
Predicted average marginal probability of infection with *Eimeria* at a range of standardized values of the identified environmental risk factors

The results of the final multivariable logistic regression model selected using purposeful selection are presented in Table 2. Several environmental variables were found to be associated with the risk of *Eimeria* infection. There was a strong positive effect of both temperature and distance to flooded areas on risk of infection. There was also some evidence of a curvilinear relationship between the risk of *Eimeria* infection and the proportion of the 1 km area around a household that was classified as swamp land as well as with the sand content of the soil. The average marginal predicted probabilities of infection based on a range of values of each of these continuous variables are presented in Fig. 2. There was strong evidence of a large effect of age on risk of *Eimeria* infection, with animals older than 18 months being at substantially reduced risk. There was weaker evidence that male animals were at greater risk of *Eimeria* infection. The distance to a water body had a large confounding effect on the coefficient for temperature and was therefore retained in the model. Variance inflation factors were all below four.

**Table 2:** Outputs from the multivariable logistic regression model

Predictor	Odds ratio	95% confidence interval	*p* value
Age (>18 months)	0.3	0.21–0.43	<0.001
Male sex	1.35	0.96–1.90	0.088
Soil sand content	1.18	0.91–1.53	0.2
Soil sand content^2^	1.1	0.99–1.23	0.07
Swampy land composition	1.12	0.87–1.44	0.38
Swampy land composition^2^	0.85	0.73–1.00	0.05
Distance to water bodies	0.85	0.68–1.06	0.14
Distance to flooded areas	1.49	1.17–1.91	0.001
Temperature	1.4	1.06–1.86	0.02

### Spatial clustering

**Fig. 3:**
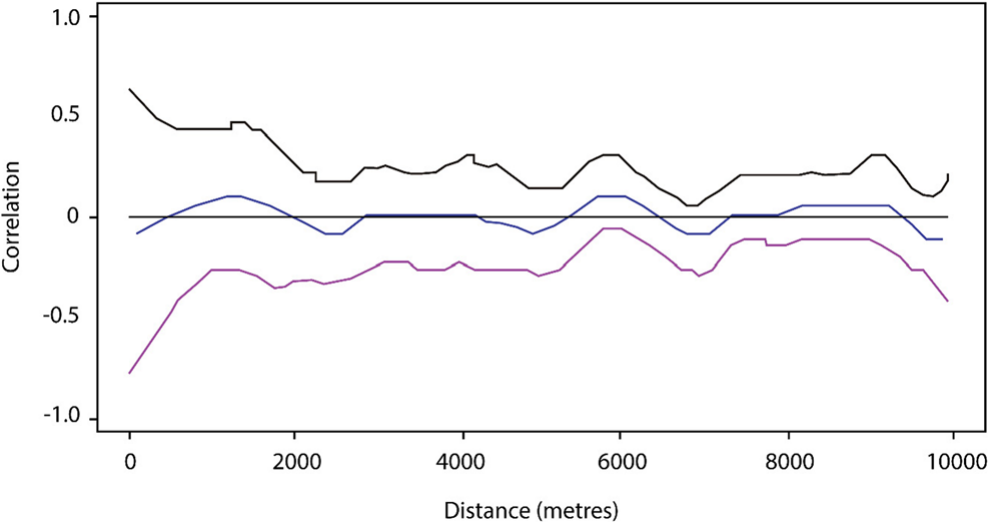
Spline correlogram comparing values in household level residuals over a range of distances

There was no evidence of clustering of high or low values of household level residuals based on a weighted normal spatial scan statistic. The 95% confidence interval for the spline correlogram included zero up to distances of 10000 m, indicating no evidence of residual spatial correlation in household level residuals (Fig. 3).

## Discussion

This study explores the prevalence of bovine infection by *Eimeria* species and associated risk factors in western Kenya, in a tropical region broadly representative of smallholder livestock production systems in the Lake Victoria basin. The overall prevalence observed was 32.7% (95% CI 29.7–35.7) while the prevalence in calves (<18 months) was 48.2% (95% CI 41.8–54.6). The observed prevalence was lower than what has been documented in other studies in similarly extensive and semi-intensive production systems. Munyua and Ngotho (1990) estimated the prevalence of bovine *Eimeria* infection in Kenya at 67.4%. Pandit (2009) recorded a prevalence of 75.8% in calves in extensive and semi-intensive production systems in Kashmir valley in India while Asfaw et al. (2016) reported a prevalence of 71.7% in calves kept in semi-intensive systems in Ethiopia.

In this study, age was the only individual animal level factor for which there was strong evidence for an effect on *Eimeria* prevalence, with animals older than 18 months being less likely to be infected. This finding has been widely observed (Lassen et al. 2009; Bangoura et al. 2012; Dong et al. 2012; Rehman et al. 2012), and it is likely that an age-related immune effect occurs for this parasite (Fiege et al. 1992). Protective immunity is generally dose dependent and is usually against infection with a homologous species (Rose 1971).

Outdoor husbandry management practices have been documented to reduce the risk of *Eimeria* infection in cattle (McAllister 2007; Rehman et al. 2011). This is expected to be related to differences in the survival of the oocyst in outdoor and indoor environments, as well as the effects of animal density and levels of environmental contamination, which are likely to be highest in closed housing systems (Asfaw et al. 2016; Khan et al. 2013). Animal stress, which tends to be higher in housed animals, may also be important influence on shedding (Priti et al. 2008). In our study, there was no evidence of an association between infection and husbandry practices. However, we observed that very few animals were routinely housed during the day or night: only 0.6% of the study population were reported to be managed under zero grazing systems throughout the whole year.

Various environmental factors were identified as being associated with *Eimeria* infection in cattle in the study area. In particular, there was a significant positive correlation between infection risk and increasing temperature. Warm environments are known to be favourable for the propagation of *Eimeria* parasites, with schizogony and sporogony occurring in warm, humid environments with adequate oxygen (Coetzer and Justin 2004). It is noteworthy that we were able to demonstrate an effect of very small temperature differences on risk of infection, suggesting these small temperature changes affect either the rate of development of oocysts and/or the duration of survival in the environment, which also impact on infection rates in animals.

Lower risk of infection was observed in individual cattle living in households that were closer to areas at risk of flooding. Such an effect could be attributed to the fact that flooded soils may be poorly aerated, with lower oxygen concentrations in the soil discouraging survival of oocysts or affecting the rate of development. For the successful completion of schizogony and sporogony, adequate oxygen concentration in the soil is needed (Coetzer and Justin 2004). Additionally, communal grazing areas that are prone to flooding may be avoided by farmers, reducing the stocking density and potential contamination of such environments for animals that live close to them.

Paradoxically, there was weak evidence of a positive association between *Eimeria* infection and the proportion of land surrounding a household that was covered by swamp, however, this relationship was not linear. The probability of infection increased up to almost 40% when 10% of the area around a household was classified as swamp, before decreasing to almost zero when more than 30% of the area was classified as swamp. This suggests that a small amount of swamp land creates a favourable microclimate for oocyst development, possibly through effects on humidity, but oocsyt survival or development, and animal risk, decreases once a threshold of soil moisture is reached.

Whilst we didn’t observe an effect of rainfall in the previous 30 days in this study, the peak prevalence of *Eimeria* infections in cattle in an earlier study in Pakistan was observed in the rainy and post rainy seasons (Rehman et al. 2012). Increased infection risk during rainy season could be expected to occur through contamination of pasture by the parasites spreading from other areas by surface water or through humidity associated effects on oocyst survival and development (Marquardt et al. 1960). The lack of evidence of an effect observed in this study may have been due to the fact that the nearest weather station with reliable data was Kisumu, which is more than 100 km from the study region and closer to Lake Victoria., which tends to have higher levels of rainfall than areas further from the lake (Kenya meteorological service 2015).

There was some weak evidence of a trend towards increased risk of *Eimeria* infection with increasing levels of sandy soil around a household. The relationship was curvilinear, with risk of infection appearing to initially decrease as sand content increases followed by an increase in risk at relatively higher sand contents. Sandy soils have macro-pores which allow water to percolate and are rarely waterlogged (Royal Horticultural Society 2016). Their moisture levels are usually low compared to other soil types, especially clay soils (Bruand et al. 2005). Whilst the evidence is very weak (*p* = >0.05), the shape of the relationship observed may provide further support to that described above for a possible threshold effect of soil moisture on oocyst development and survival. Such an effect warrants further exploration, particularly since land management practices, such as the implementation of drainage, can influence soil moisture content (Skaggs et al. 2006) and may impact on the risk of *Eimeria* infection.

Although eimeriosis can be a severe disease in cattle, subclinical infection is a common finding, including in temperate settings (Cornelissen et al. 1995; Farkas et al. 2007). Importantly, subclinical *Eimeria* infection has been associated with economic losses associated with low productivity and fertility in animals (Dedrickson 2000; Kristjanson et al. 2004; Maas 2007; Lassen 2009). A potential limitation of this study is that individual *Eimeria* species identification was not done, and different species could have different pathogenicity.

The prevalence of *Eimeria* infection in cattle in western Kenya is lower than has been recorded in other parts of the country and the world in similar production systems. While most infections are likely to be subclinical, farmers in western Kenya could consider laboratory diagnosis and treatment of positive cases to reduce production losses associated with infection. The prevalence of *Eimeria* in cattle in western Kenya is influenced by animal age and the temperature. The soil water content in the areas around households also appears to be important. Knowledge of these animal and environmental predictors could assist with the targeting of testing, or observation for clinical disease, to particular animals living in particular environmental conditions.
